# Molecular Mechanisms That Underlie the Dynamic Adaptation of Innate Monocyte Memory to Varying Stimulant Strength of TLR Ligands

**DOI:** 10.3389/fimmu.2016.00497

**Published:** 2016-11-10

**Authors:** Ruoxi Yuan, Shuo Geng, Liwu Li

**Affiliations:** ^1^Department of Biological Sciences, Virginia Polytechnic Institute and State University, Blacksburg, VA, USA; ^2^Department of Biomedical Sciences and Pathobiology, Virginia–Maryland College of Veterinary Medicine, Virginia Polytechnic Institute and State University, Blacksburg, VA, USA

**Keywords:** dynamic adaptation, innate monocyte, memory, signal strength, mechanisms

## Abstract

In adaptation to rising stimulant strength, innate monocytes can be dynamically programed to preferentially express either pro- or anti-inflammatory mediators. Such dynamic innate adaptation or programing may bear profound relevance in host health and disease. However, molecular mechanisms that govern innate adaptation to varying strength of stimulants are not well understood. Using lipopolysaccharide (LPS), the model stimulant of toll-like-receptor 4 (TLR4), we reported that the expressions of pro-inflammatory mediators are preferentially sustained in monocytes adapted by lower doses of LPS, and suppressed/tolerized in monocytes adapted by higher doses of LPS. Mechanistically, monocytes adapted by super-low dose LPS exhibited higher levels of transcription factor, interferon regulatory factor 5 (IRF5), and reduced levels of transcriptional modulator B lymphocyte-induced maturation protein-1 (Blimp-1). Intriguingly, the inflammatory monocyte adaptation by super-low dose LPS is dependent upon TRAM/TRIF but not MyD88. Similar to LPS, we also observed biphasic inflammatory adaptation and tolerance in monocytes challenged with varying dosages of TLR7 agonist. In sharp contrast, rising doses of TLR3 agonist preferentially caused inflammatory adaptation without inducing tolerance. At the molecular level, the differential regulation of IRF5 and Blimp-1 coincides with unique monocyte adaptation dynamics by TLR4/7 and TLR3 agonists. Our study provides novel clue toward the understanding of monocyte adaptation and memory toward distinct TLR ligands.

## Introduction

Emerging studies suggest that innate leukocytes may adopt “rudimentary” memory states depending on challenge history and strength, as reflected in the priming and tolerance paradigm of monocytes challenged with varying dosages of bacterial endotoxin lipopolysaccharide (LPS) (1). LPS is a membrane component of common mucosa gram-negative bacteria. Upon leakage into host systemic circulation, higher dosages of LPS may elicit a strong, yet, transient inflammatory cytokine storm followed by suppression of inflammation and tolerance (2). Both the dramatic upswing of inflammatory cytokines as well as the late-phase refractory tolerance contribute to severe morbidity and mortality associated with sepsis (3). In sharp contrast, subclinical leakage of super-low dose LPS may occur in humans with chronic low-grade inflammatory disease (4, 5). Monocytes with prolonged adaptation to super-low dose LPS fail to develop tolerance and give rise to a non-resolving low-grade inflammatory phenotype conducive for chronic inflammatory disease (6). However, differential monocyte adaptations to challenges by other microbial products have not been well studied.

Upon microbial challenges, toll-like-receptors (TLRs) modulate complex plethora of signaling molecules that eventually activate both transcriptional activators and suppressors of inflammatory mediators. Of particular significance, interferon regulatory factor 5 (IRF5) has been a recognized master transcription factor of pro-inflammatory monocytes and was shown to induce expression of pro-inflammatory genes, such as IL-12 and IL-23, in both murine and human inflammatory monocytes (7). On the other hand, B lymphocyte-induced maturation protein-1 (Blimp-1) has been reported to be a critical transcriptional repressor of inflammatory genes. Blimp-1 may also be involved in the induction of anti-inflammatory mediators (8). In addition, Blimp-1 is essential for modulating homeostasis of NK cell (9), T cell (10), and dendritic cell (11). Blimp-1 also contributes to the homeostatic regulation of bone (12) and intestinal tissues (13). However, the modulations of IRF5 and Blimp-1 by varying dosage of LPS and their connection to monocyte adaptation have not been well examined.

Emerging data and computational analyses suggest that the potential competition among multiple signaling pathways within monocytes may be responsible for the dynamic adaptation of monocytes (14). In the context of TLR signaling processes, there are at least two potentially competitive pathways, namely MyD88-dependent and MyD88-independent pathways (15). MyD88 is a critical adaptor molecule that directs signaling traffic within innate monocytes. Other key adaptors include TRIF and TRAM (16). Previous studies suggest that MyD88 and TRIF were involved in high dose LPS induced endotoxin tolerance effects (17). However, little is known about the roles of other adaptor molecules, especially TRAM, which was shown to be pro-inflammatory and pro-atherogenic during the pathogenesis of atherosclerosis (18).

To fill the critical void in this intriguing area of innate monocyte adaptation and memory, we examined the differential adaptation of monocytes by varying dosages of LPS and potential underlying mechanisms. We characterized the expression profiles of selected pro- and anti-inflammatory mediators in murine monocytes challenged with varying dosages of LPS as well as TLR3/7 agonists. The dynamic expression profiles of inflammatory mediators were cross-examined with key transcriptional modulators such as IRF5 and Blimp-1. The potential involvement of MyD88 and TRAM/TRIF during the dynamic adaptation of monocytes were studied by employing primary bone marrow monocytes (BMM) collected from wild-type (WT), MyD88^−/−^, TRIF^−/−^, and TRAM^−/−^ mice.

## Results

### Monocytes Adapted to Higher Dose LPS Exhibit Inflammatory Tolerance While Preferentially Express Homeostatic Mediators. In Contrast, Monocytes Adapted to Super-Low Dose LPS Develop a Low-grade Inflammatory Profile without the Expression of Homeostatic Mediators

**Figure 1:**
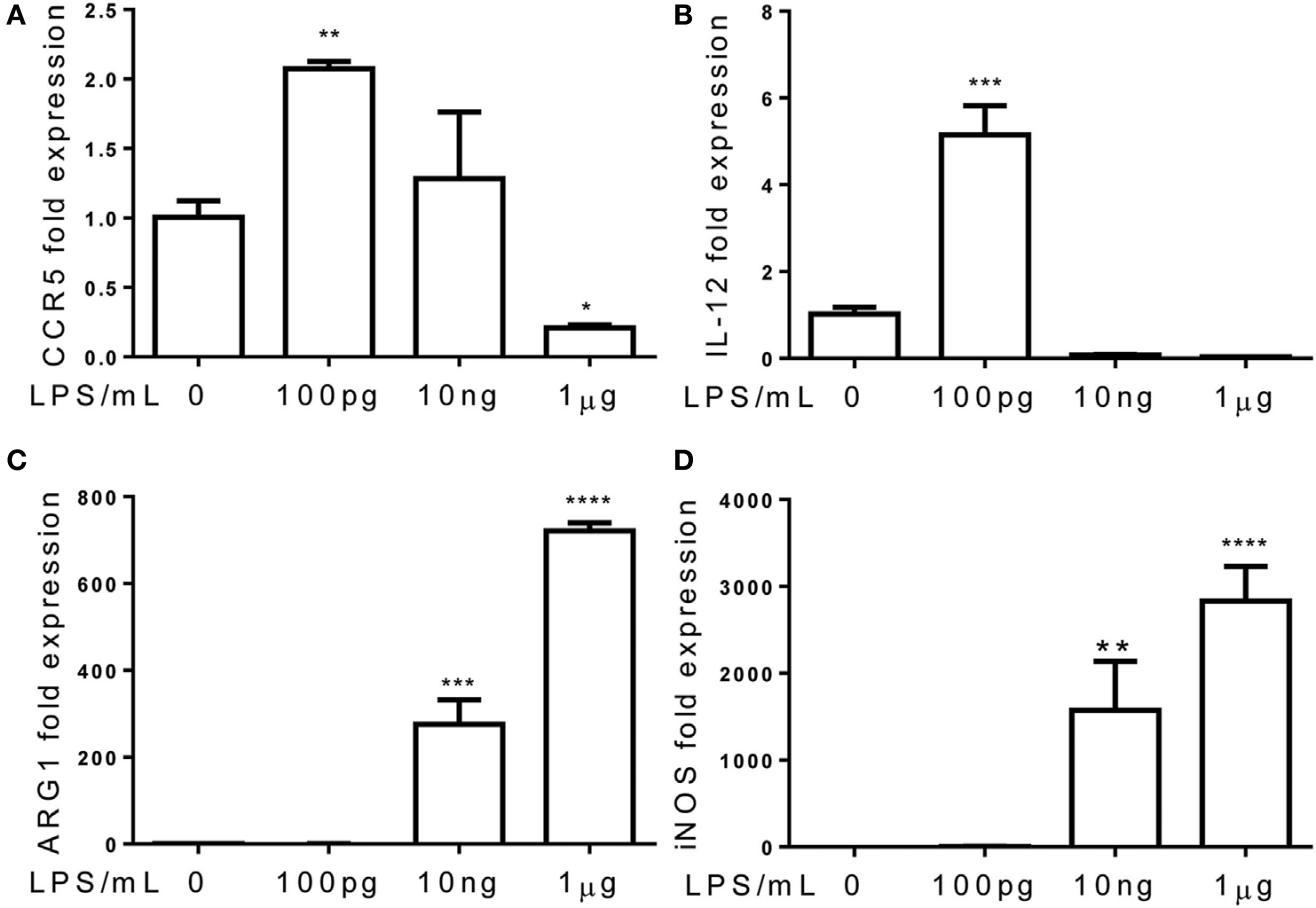
**Differential priming and tolerance by TLR agonists**. Total RNA was isolated from monocytes treated with different dosages of LPS for 5 days. Real-time PCR was performed to determine the expression levels of CCR5 **(A)**, IL-12 **(B)**, ARG1 **(C)**, and iNOS **(D)**. Data are representative of three separate experiments (error bar represent SEM, **p < 0.01, ***p < 0.001, ****p < 0.0005, as compared to non-treated control group, Student’s *t*-test).

**Figure 2:**
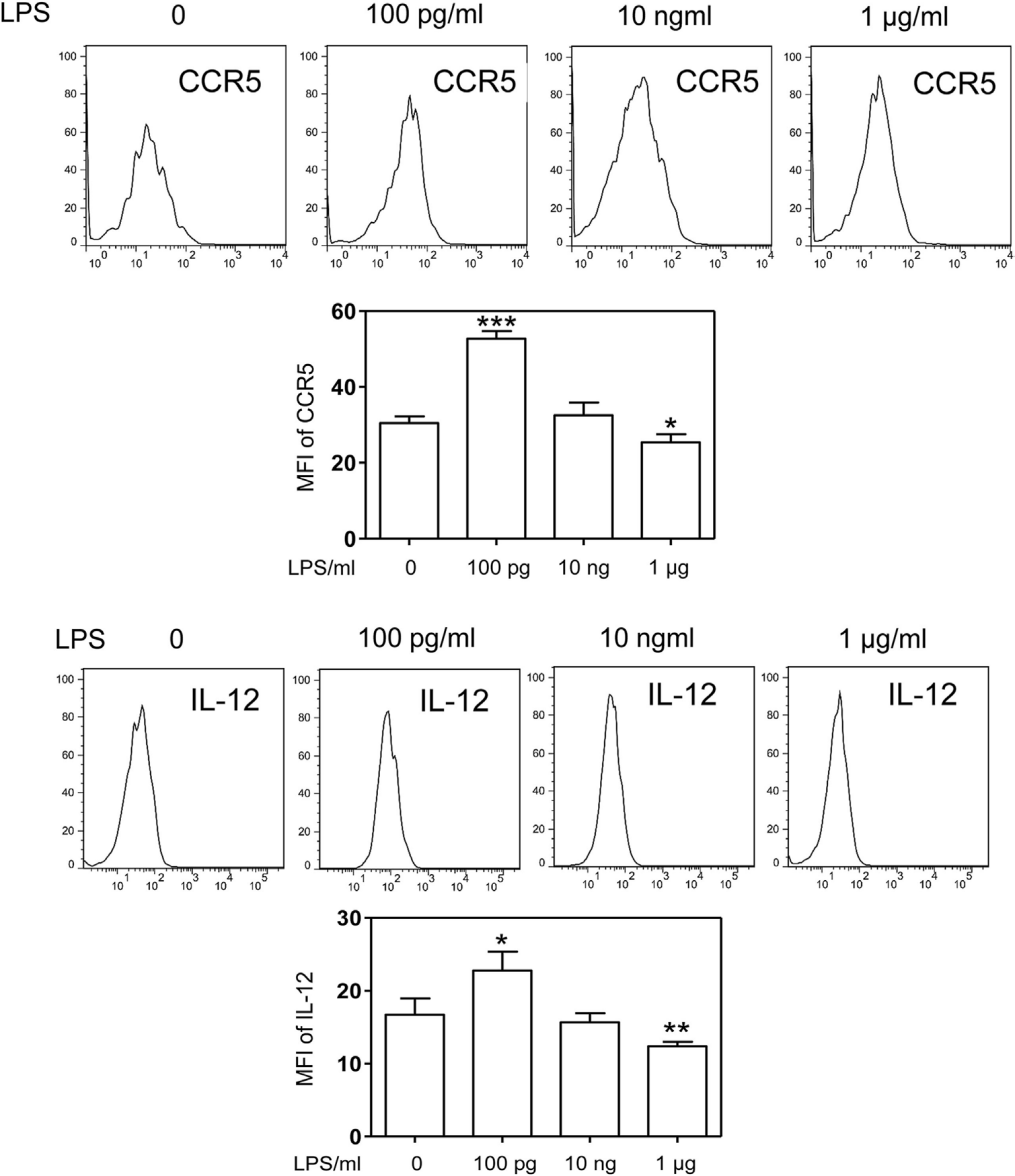
**Differential modulation of CCR5 and IL-12 protein levels by LPS**. Differentially challenged cells were immunostained with specific antibodies against either CCR5 or IL-12, and cellular protein levels of CCR5 and IL-12 were monitored by flow cytometry. Error bar represents SEM of three experiments. *p < 0.05, **p < 0.01, ***p < 0.001, as compared to non-treated control group, Student’s *t*-test.

Although the phenomena of endotoxin tolerance and priming triggered by a short period of single dose LPS challenge have been well documented (19–21), limited studies are performed to characterize the adaptation effects of prolonged LPS exposures on monocytes. In this context, we assessed the gene expression profiles of murine monocytes continuously challenged with varying dosages of LPS for a 5-day period. We checked the cell viability among treated cells and observed similar cell survival among 5-day cultures of cells challenged varying dosages of LPS (Figure S1 in Supplementary Material). We also checked the cell surface expression of CD11b, a key marker for monocytes, and observed that >99% of living cells cultured by M-CSF for the 5-day period are CD11b positive (Figure S2 in Supplementary Material). As shown in Figure 1, monocytes were adapted into an anti-inflammatory “tolerant” phenotype after a prolonged challenge with higher dose LPS (1 μg/ml), as reflected in a significant reduction of selected pro-inflammatory genes such as IL-12 and CCR5 (Figures 1A,B), resembling the phenotype of endotoxin tolerance. Higher dose LPS-adapted monocytes become potent producers of homeostatic genes involved in tissue repair such as ARG1 and iNOS (Figures 1C,D). In sharp contrast, monocytes challenged with super-low dose LPS (100 pg/ml) were adapted to express significantly higher levels of inflammatory mediators (IL-12 and CCR5) as compared to either non-adapted monocytes or high dose LPS-adapted monocytes (Figures 1A,B; Figure S3 in Supplementary Material). We confirmed the protein levels of selected targets such as IL-12 and CCR5 through flow analyses (Figure 2). The super-low dose LPS-adapted monocytes did not express homeostatic tissue-repair genes ARG1 or iNOS. These data reveal distinct adaptation of monocytes into either a non-resolving inflammatory state or resolving tolerant state, dependent upon the relative signal strengths of prolonged LPS challenges.

The signature transcription factor of inflammatory monocyte IRF5 is induced in monocytes adapted by super-low dose LPS, and drastically reduced in monocytes adapted by higher dose LPS. On the other hand, the transcriptional modulator Blimp-1 is reduced in monocytes adapted by super-low dose LPS and elevated in higher dose LPS-adapted monocytes.

**Figure 3:**
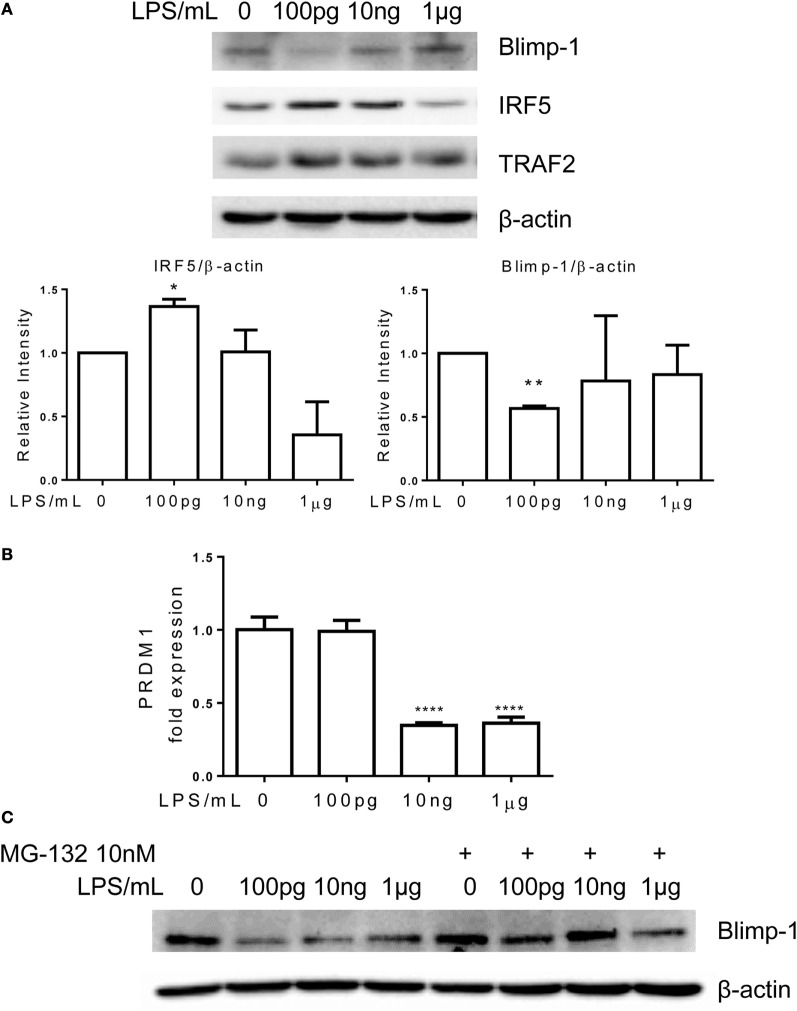
**Differential regulation of IRF5 and PRDM1 by different dosages of LPS**. Monocytes were treated with different dosages of LPS w/o MG132 as indicated, total RNA was isolated for RT-PCR **(B)**, and whole cell lysates were obtained for western blot. Proteins were separated on SDS-PAGE and levels of Blimp-1, IRF5, TRAF2, and β-actin were detected **(A,C)**. Data are representative of three separate experiments. Error bars represent SEM of three experiments. *p < 0.05, **p < 0.01, ***p < 0.001, as compared to non-treated control group, Student’s *t*-test.

Interferon regulatory factor 5 is a key signature transcription factor within inflammatory monocytes (7, 22), while Blimp-1 serves as an important homeostatic modulator in myeloid cells (23). Elevation of IRF5 or reduction of Blimp-1 were associated with the pathogenesis of chronic inflammatory diseases (8, 24, 25). Together with other transcriptional activators, IRF5 activates the expression of inflammatory mediators. On the other hand, Blimp-1 may serve as an anti-inflammatory mediator through competing and inhibiting target sequences of IRFs (26). To determine the molecular mechanisms responsible for the dynamic adaptation of monocytes, we examined the cellular levels of IRF5 and Blimp-1. Consistent with the tolerant phenotype, monocytes adapted with higher dose LPS (1 μg/ml) had markedly reduced levels of IRF5 and restored Blimp-1 levels as compared to control monocytes (Figure 3A). In sharp contrast, the non-resolving inflammatory monocytes adapted with 100 pg/ml LPS had elevated levels of IRF5 and reduced levels of Blimp-1 (Figure 3A).

The reduction of inflammatory suppressor Blimp-1 may allow for the development of non-resolving inflammatory monocyte. We further tested whether reduced gene expression or protein stability may account for Blimp-1 reduction in non-resolving inflammatory monocytes. As shown in Figure 3B, the mRNA levels of PRDM1, the gene encoding Blimp-1, were not altered comparing control monocytes and monocytes adapted by 100 pg/ml LPS. This suggests that Blimp-1 reduction in adapted inflammatory monocytes may be caused by reduced protein stability. To confirm this, we applied proteasome inhibitor MG-132. As shown in Figure 3C, application of MG-132 abolished the reduction of Blimp-1 in monocytes adapted by lower dose LPS. The degradation of Blimp-1 has not been studied in monocytes, although a previous report suggested that TNF receptor-associated factor 2 (TRAF2)-mediated JNK activation may be critically involved in Blimp-1 downregulation in B cells (27). Previous reports revealed that TRAF2 overexpression was correlated with upregulated expression of inflammatory genes (28, 29). In addition, TRAF2 also plays central role in signaling pathway induced by ER stress (30, 31). Thus, we further tested the levels of TRAF2 in differentially adapted monocytes. We observed that TRAF2 levels were induced in non-resolving inflammatory monocytes adapted by super-low dose LPS (Figure 3A). In contrast, TRAF2 levels returned to resting levels in tolerant monocytes adapted by 1 μg/ml LPS (Figure 3A).

**Figure 4:**
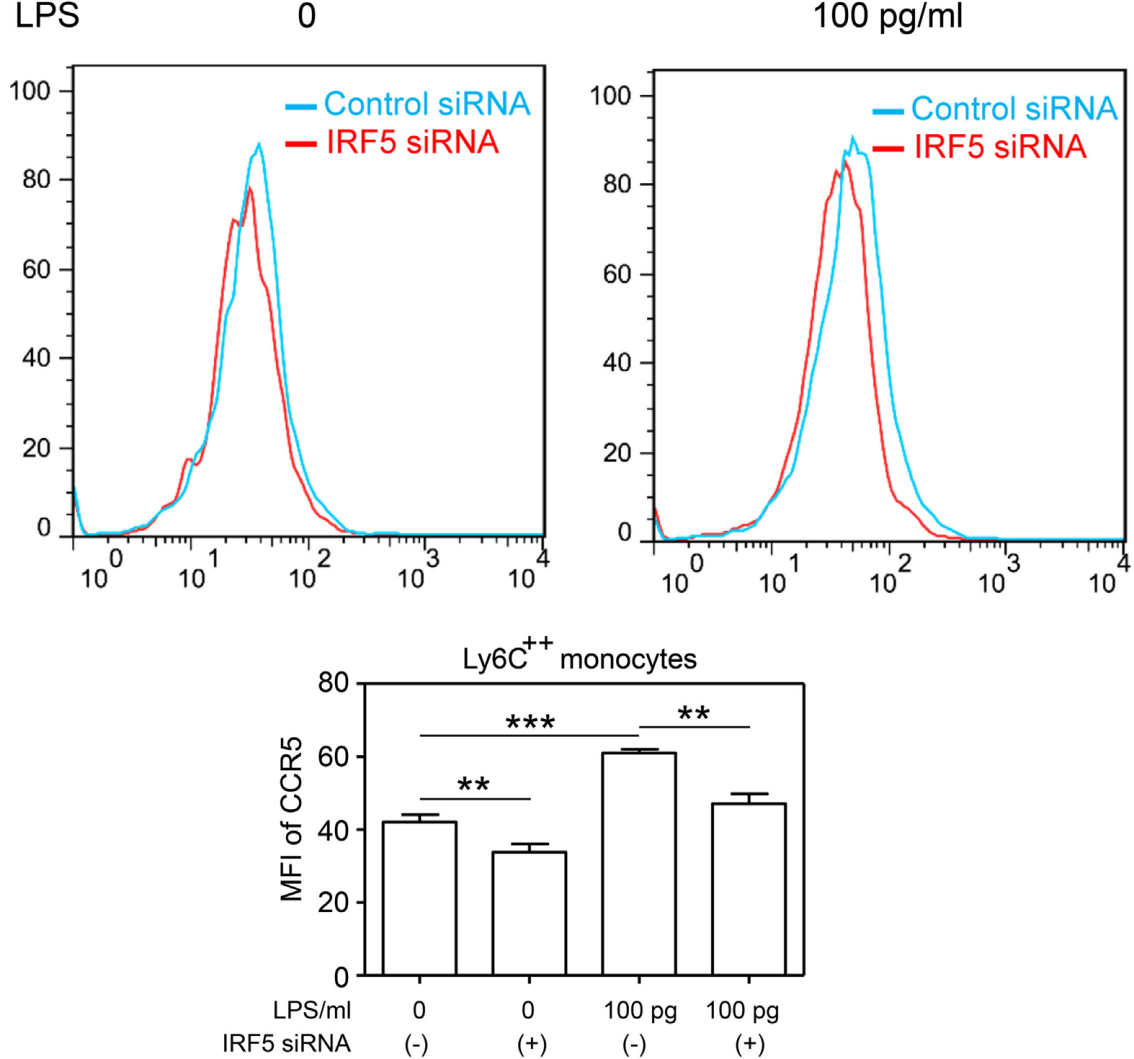
**Selective knockdown of IRF5 abolishes the induction CCR5 by super-low dose LPS monocytes co-cultured with either control or IRF5-specific siRNAs were challenged with LPS**. The levels of CCR5 were measured by flow cytometry. Error bar represents SEM of three experiments. **p < 0.01, ***p < 0.001, as compared to non-treated control group, Student’s *t*-test.

To further provide causative proof with regard to the role of IRF5 in the programing of monocytes, we performed siRNA analyses to selectively decrease the levels of IRF5. We observed that selective knockdown of IRF5 led to the abolishment of CCR5 induction by super-low dose LPS (Figure 4).

### The Non-Resolving Inflammatory Monocyte Adaptation Is Independent of MyD88, but Dependent on TRAM and TRIF

**Figure 5:**
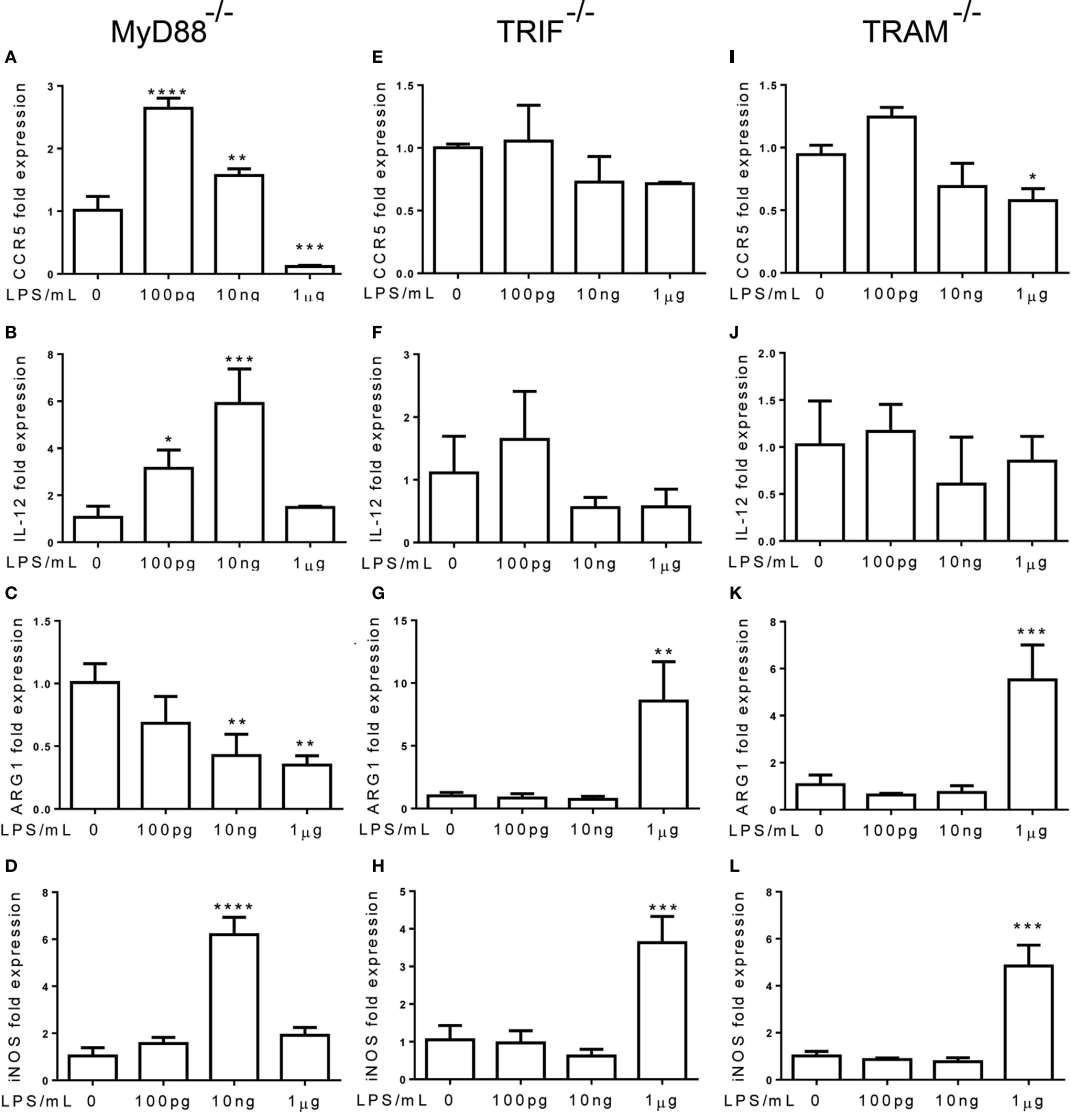
**The non-resolving inflammatory monocyte adaptation is independent of MyD88, but dependent on TRAM and TRIF**. **(A–L)** Total RNAs were harvested from LPS-treated monocytes with MyD88, TRIF, and TRAM deficiency. Real-time PCR was performed to determine the expression levels of CCR5 **(A,E,L)**, IL-12 **(B,F,J)**, ARG1 **(C,G,K)**, and iNOS **(D,H,L)**. Data are representative of three separate experiments (error bar represent SEM, *p < 0.05, **p < 0.01, ***p < 0.001, ****p < 0.0001, as compared to non-treated control group, Student’s *t*-test).

Next, we tested the roles of TLR adaptor molecules during the dynamic adaptation of monocytes. Bone marrow monocytes from MyD88^−/−^ and TRIF^−/−^ mice were adapted for 5 days with super-low and higher dosages of LPS. As shown in Figures 5A,B, the induction of inflammatory mediators, such as IL-12 and CCR5, was not affected by MyD88 deficiency, suggesting that MyD88 is not involved in the inflammatory adaptation of monocytes. In contrast, the induction of inflammatory IL-12 and CCR5 was abolished in TRIF or TRAM-deficient monocytes (Figures 5E,F,I,J). On the other hand, the homeostatic monocyte adaptation to higher dose LPS as reflected in the expression of ARG1 was dependent upon MyD88, but not TRIF or TRAM (Figures 5C,G,K). TRIF or TRAM deletion did not affect the induction of iNOS by higher dose LPS (Figures 5D,H,L). Our data further support the emerging concept that the adaptation processes of monocytes are highly dynamic, complex, and may differentially involve unique signaling pathways. In terms of the non-resolving inflammatory monocyte adaptation, TRAM and TRIF, instead of MyD88 are critically required.

**Figure 6:**
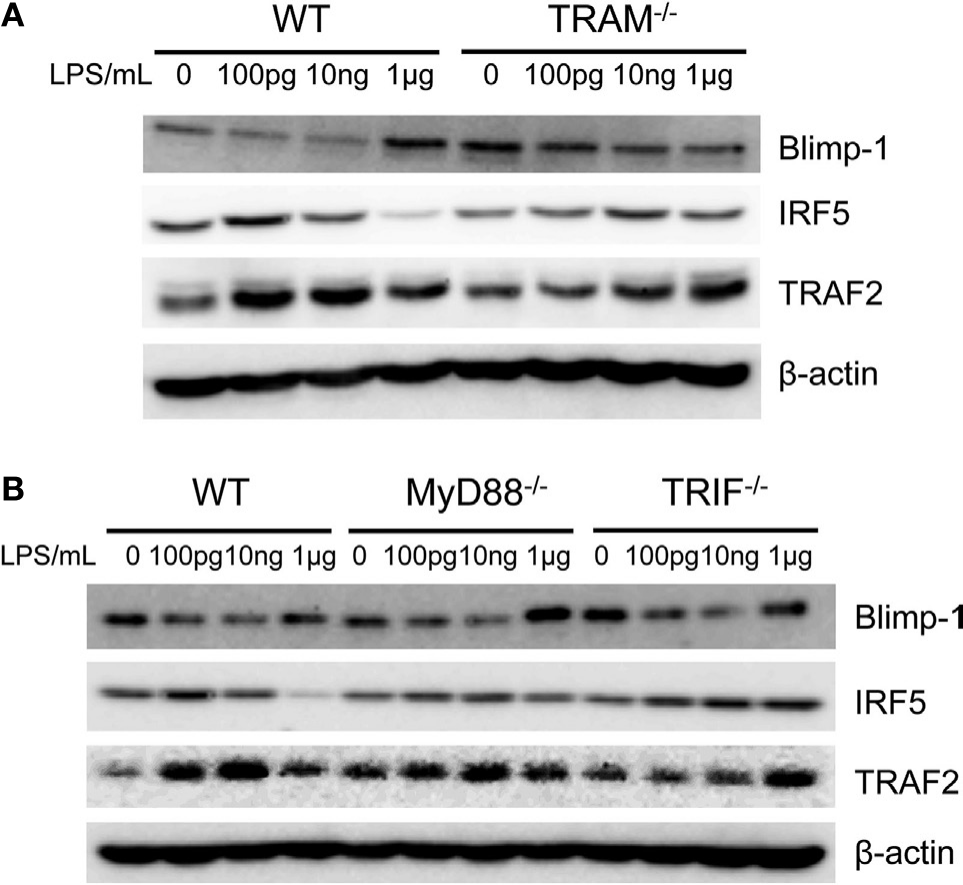
**Differential modulation of IRF5 and Blimp-1**. Whole cell lysates were harvested from LPS-treated monocytes with MyD88, TRIF, and TRAM deficiency. Protein levels of Blimp-1, IRF5, TRAF2, and β-actin were detected **(A,B)**. Data are representative of three separate experiments.

Downstream of TRAM/TRIF, we further tested whether TRAM or TRIF may be involved in the activation of IRF5, TRAF2, and reduction of Blimp-1 in monocytes adapted by super-low dose LPS. As shown in Figure 6A, the induction of TRAF2, IRF5, and the reduction of Blimp-1 in inflammatory monocytes adapted by super-low dose LPS (100 pg/ml) were abolished in TRAM-deficient BMDM. On the other hand, MyD88-deficient BMM adapted by 100 pg/ml LPS experienced similar induction of TRAF2, IRF5, and reduction of Blimp-1 as compared to WT BMM (Figure 6B). Compared with WT monocytes, the induction of TRAF2 in TRIF-deficient monocytes was attenuated. Taken together, our data suggest that the non-resolving inflammatory adaptation of monocytes to super-low dose LPS is not MyD88 dependent, and dependent on TRAM/TRIF pathway. Our results are consistent with previous findings of pro-inflammatory role of TRAM/TRIF in the chronic pathogenesis of atherosclerosis (18).

### TLR7 Agonist Induces Both Inflammatory and Tolerant Adaptation of Monocyte, While TLR3 Agonist Selectively Induces Inflammatory Adaptation without Inducing Monocyte Tolerance

**Figure 7:**
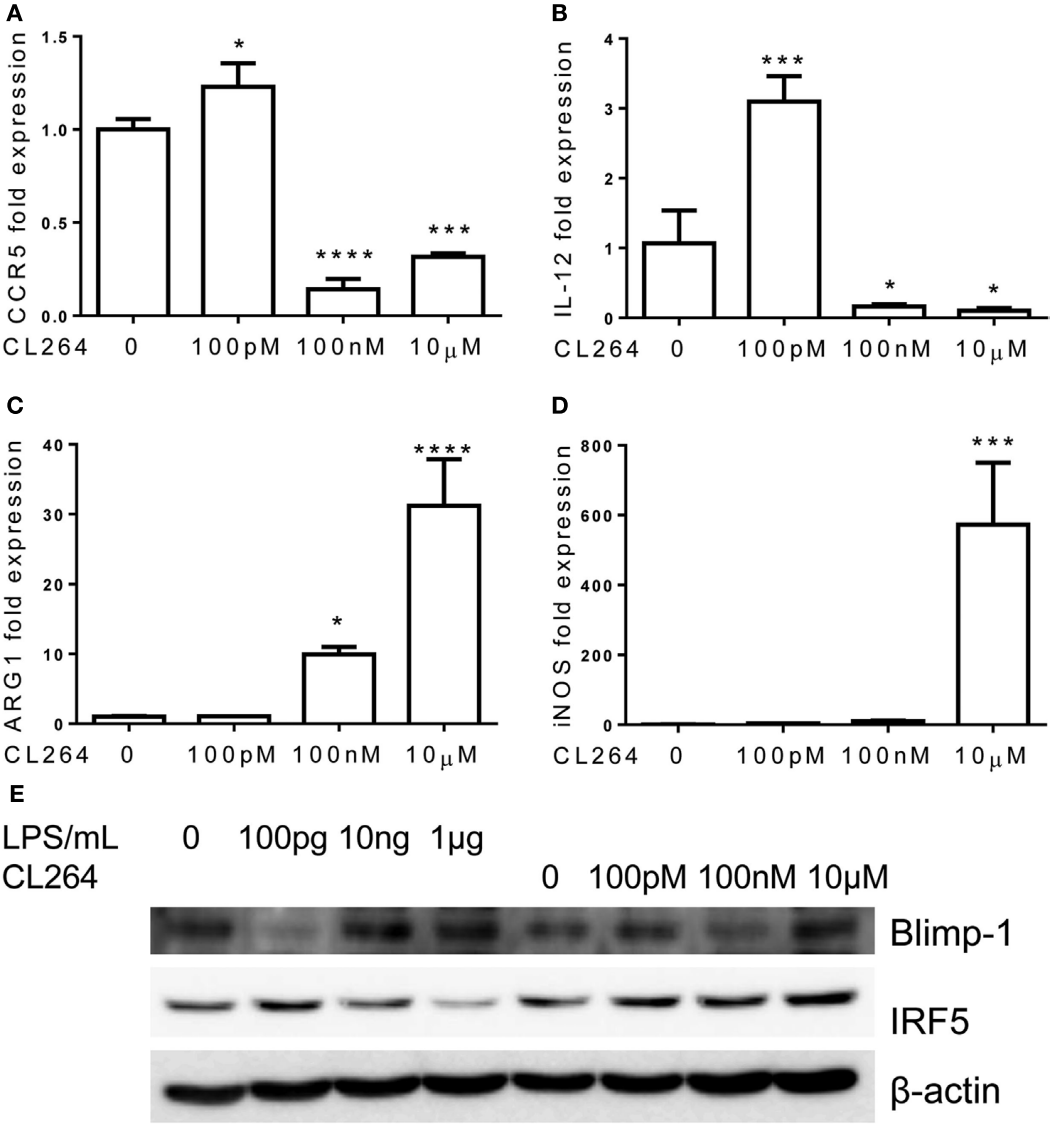
**Differential regulation of monocytes responses by TLR7 ligand CL264**. Total RNA was isolated from monocytes treated with different dosages of CL264 for 5 days. Real-time PCR was performed to determine the expression levels of CCR5 **(A)**, IL-12 **(B)**, ARG1 **(C)**, and iNOS **(D)**. Protein levels of Blimp-1, IRF5, and β-actin were determinate by western blot **(E)**. Data are representative of three separate experiments (error bar represent SEM, *p < 0.05, ***p < 0.001, ****p < 0.0001, as compared to non-treated control group, Student’s *t*-test).

Toll-like receptor 4 is unique among TLRs for its usage of both MyD88 and TRAM/TRIF-dependent pathways. Other TLRs may preferentially use one of these pathways. This led us to explore whether the inflammatory adaptation of monocytes is limited to TLR4 and LPS signaling pathway or may be similarly programed by other TLR agonists. To this regard, we specifically tested the effects of TLR3 and TLR7 agonists, given the fact that TLR3 selectively uses TRIF-dependent pathway and that TLR7 may use both the MyD88 and TRAM pathways (16, 32). Indeed, monocytes adapted by varying dosages of TLR7 agonist CL264 exhibited similar dynamics as compared to monocytes adapted by LPS. Monocytes with prolonged challenges with lower dose of TLR7 agonist CL264 (100 nM) were selectively adapted into an inflammatory state with elevated expressions of IL-12 and CCR5, and no expression of Arg1 and iNOS (Figures 7A–D). In contrast, monocytes adapted with higher dose CL264 (100 nM, 1 μM) were tolerant with reduced expression of IL-12 and CCR5, and elevated induction of homeostatic genes such as ARG1 and iNOS.

In terms of the molecular mechanisms, we observed partially similar patterns of IRF5 and Blimp-1 modulation by CL-264 as compared to LPS. Lower-dose CL-264 induced IRF5 and reduced Blimp-1. In contrast, although failed to reduce IRF5 level, higher dose CL-264 restored Blimp-1 in adapted monocytes (Figure 7E).

**Figure 8:**
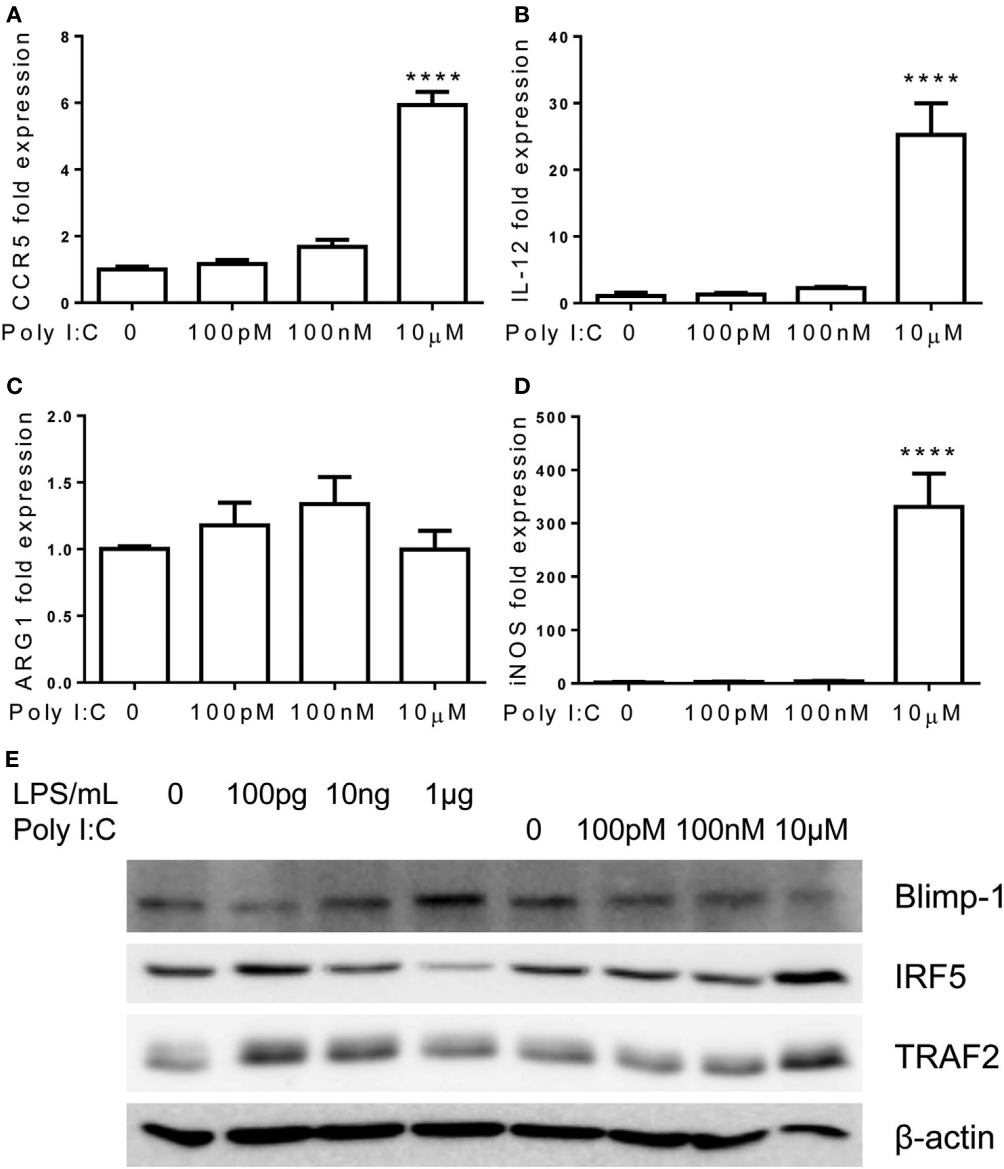
**Differential regulation of monocytes responses by TLR3 ligand Poly I:C**. Total RNA was isolated from monocytes treated with different dosages of Poly I:C for 5 days. Real-time PCR was performed to determine the expression levels of CCR5 **(A)**, IL-12 **(B)**, ARG1 **(C)**, and iNOS **(D)**. Protein levels of Blimp-1, IRF5, TRAF2, and β-actin were determinate by western blot **(E)**. Data are representative of three separate experiments (error bar represent SEM, ****p < 0.0001, as compared to non-treated control group, Student’s *t*-test).

In contrast to TLR7/4 agonists, monocytes failed to develop tolerant adaptation when challenged with TLR3 agonist Poly I:C (Figure 8). The expression of pro-inflammatory mediators, IL-12 and CCR5, kept rising with increasing amount of Poly I:C challenges, and ARG1 was not induced by higher dosages of Poly I:C (Figures 8A–C). At the mechanistic level, the inductions of IRF5 and TRAF2 were unabated by rising concentrations of Poly I:C (Figure 8E). Rising concentrations of Poly I:C also led to a persistent reduction of Blimp-1 without restoration as compared to adapted monocytes by higher dose of LPS challenge (Figure 8E). Taken together, our data reveal that TLR7 agonist induces similar inflammatory monocyte adaptation and tolerance, as elicited by LPS. In contrast, TLR3 agonist preferentially induces inflammatory adaptation without tolerance.

## Discussion

Our data collected from this study offer novel insight with regard to the adaptation dynamics of innate monocytes. We observed that TLR4 agonist LPS may adapt monocytes into either a non-resolving inflammatory state or a tolerance anti-inflammatory state depending upon LPS signal strength. Prolonged monocyte adaptation to higher dosages of LPS led to reduced expression of pro-inflammatory mediators such as IL-12 and CCR5, and elevated expression of anti-inflammatory mediators such as ARG1 and iNOS. In contrast, prolonged adaptation to super-low dose LPS gave rise to an opposite phenotype with elevated expression of IL-12/CCR5. Lower dose LPS may favor the inflammatory monocyte adaptation *via* the activation of IRF5 and reduction of Blimp-1 through TRAM/TRIF, instead of MyD88, mediated pathway. Resembling TLR4, TLR7 agonist may similarly modulate the dynamic monocyte adaptation, with lower dosages of CL264 inducing, while higher dosages of CL264 reducing the expression of inflammatory mediators IL-12/CCR5. On the other hand, TLR3 agonist Poly I:C exhibited uniphasic adaptation of pro-inflammatory monocytes. Together, our data demonstrated the potential presence of competing circuitries that can fine tune monocyte memory dynamics in adaptation to varying TLR signal strength.

Monocyte adaptation reflects a unique facet of the emerging innate memory concept (21, 33). Previous attempts at defining innate memory have largely limited to the leukocyte phenotypic modifications due to challenges with distinct stimulants, as reflected in the distinct macrophage phenotypes programed by either IFN-γ or IL-4 (34). Limited attention has been given to signal strength-dependent programing on innate leukocytes. Reports from us and others indicated that LPS can dose-dependently induce either monocyte/macrophage priming or tolerance to a subsequent higher dose LPS challenge (6, 21, 35). This current study expands these previous findings and demonstrates that prolonged stimulation of innate monocyte with lower-dose LPS drives an inflammatory phenotype, while prolonged stimulation with higher-dose LPS causes an anti-inflammatory adaptation. We further demonstrate that IRF5 is a critical transcription factor involved in the enhanced expression of IL-12 and CCR5 in programed monocytes by super-low dose LPS. We further observed that monocytes may differentially adapt to prolonged challenges of unique TLR agonists such as TLR7 and TLR3 agonists. Our data may bear significant conceptual and translational implications. In contrast to the simple paradigm of one signal accounting for one phenotype that is a far-cry from the complex dynamics of monocyte memory, our data reveal dynamic adaptation of innate monocytes dependent upon not only the chemical nature of corresponding stimulant but also the signal strength and history of stimulation. In line with our finding, a recent report demonstrates opposing effects of acute and chronic administration of TGF-β on immune cell activation (36). Our current study provides a conceptual clue, which warrants future systems analyses of complex dynamics of monocyte adaptation.

At the mechanistic level, our data clarify an important issue with regard to the differential usages of MyD88 and TRAM/TRIF pathways during monocyte adaptation and memory. Although initial studies with cell lines and overexpression analyses suggested that MyD88 may account for the inflammatory signaling processes downstream of TLR4 (37), *in vivo* analyses with MyD88 knockout mice suggested a much more complex scenario (38, 39). With the chronic atherosclerosis model, TRAM, or TRIF deficiency, instead of MyD88 deficiency, was found to have reduced inflammation and atherosclerosis progression (18). Recent molecular analyses reveal that MyD88 pathway not only induces NF-κB activation but also multiple negative feedback loops that cause tolerant adaptation (40, 41). In contrast, our current study was the first report revealing TRAM/TRIF path as the key circuit involved in the low-grade inflammatory adaptation of monocytes. Instead of inducing negative feedback modulators, our data suggest that TRAM/TRIF pathway may be responsible for the removal of negative modulators such as Blimp-1, thus disallowing the development of anti-inflammatory tolerance and favoring the inflammatory monocyte adaptation.

Our observation with regard to inflammatory and anti-inflammatory adaptation of monocytes to distinct TLR agonists with varying stimulant strength may hold potential translational values in therapies against chronic disease. For example, anti-inflammatory monocyte adaptation by higher TLR7 agonist may hold potential value in the treatment of chronic inflammatory disease such as atherosclerosis. Re-enforcing this consideration, a previous animal study with TLR7 agonist has shown promise in alleviating the progression of atherosclerotic plaques (42). It is intriguing to note that TLR3 agonist fails to illicit anti-inflammatory monocyte adaptation, potentially due to its sole usage of TRIF pathway instead of the MyD88 pathway. This is in consistent with some previous reports that demonstrated the lack of tolerance in monocytes/macrophages treated with TLR3 agonist. For example, a previous study with RAW264.7 cells revealed that the pretreatment with Poly I:C led to increased IL-12 production following a subsequent LPS stimulation (43, 44). At the translational level, low-grade inflammatory monocyte adaptation by TLR3 agonist may serve as a potential strategy to boost immune surveillance of tumor. Our work also re-enforces the significance of carefully considering the effects of drug dosages in the prevention and treatment of chronic inflammatory diseases.

Our current study has addressed a focused mechanism regarding the programing of monocytes. Future studies are clearly needed to systematically address the wider and complex signaling networks involved in the dynamic programing of monocytes. In addition to the differential usage of adaptors and transcription factors, distinct configurations of cellular receptors and/or co-receptors may also play a significant role. We have indeed observed that the levels of TLR4 and CD14 may vary in cells challenged with varying dosages of LPS (Figure S4 in Supplementary Material). In addition, other cellular mediators (either soluble or cell-membrane bound) produced through the training program may exert autocrine effects for the differential programing. However, our prolonged culture study may mimic the *in vivo* situation where persistent TLR agonists may exit and contribute to inflammatory polarization. Our phenotypic observations with regard to priming and tolerance also are consistent with previous studies with short-time LPS exposures (17, 21, 35, 41). Furthermore, our study also complements previous studies with human monocytes with prolonged training (33). Extensive future studies are warranted to perform fine-mapping of monocyte phenotypes in adaptation to diverse TLR agonists and antagonists, in order to harness the full potential of monocyte memory in the treatment and prevention of human disease.

## Materials and Methods

### Animals

C57BL/6 were maintained and bred under standard pathogen-free conditions. MyD88^−/−^ and TRIF^−/−^ mice were kindly provided by Dr. Michael Fessler at National Institute of Environmental Health Sciences. TRAM^−/−^ mice were generously provided by Dr. Holger Eltzschig at University of Colorado. 8- to 12-week-old male mice were used for the experiments. All animal experiments were approved, prior to the initiation of this study, by the Institutional Animal Care and Use Committee (IACUC) of Virginia Polytechnic Institute and State University.

### Reagents

Lipopolysaccharide (*Escherichia coli* 0111:B4) was purchased from Sigma. CL264 and Poly I:C were purchased from InvivoGen. Murine macrophage colony-stimulating factor (M-CSF) was obtained from PeproTech. Anti-Blimp-1 antibody, anti-IRF5, anti-TRAF2 antibodies were obtained from cell signaling technology. Anti-β-actin antibody was obtained from Santa Cruz.

### Protein Extraction and Analyses

Cells were washed with cold PBS after specified treatments and harvested in SDS lysis buffer containing protease and phosphatase inhibitors as previously described (21, 44). Protein concentration was assessed by Bradford assay. Following SDS-PAGE, protein bands were transferred to an immunoblot PVDF membrane (Bio-Rad) and subjected to immunoblot analysis with indicated antibodies. Intensity of each band was quantified using the Multi Gage software (Fujifilm).

### Real-time RT-PCR Analyses

Total RNA was extracted using TRIzol (Thermo Fisher Scientific), according to the manufacturer’s protocol. RNA was reverse-transcribed using the high-capacity cDNA reverse transcription kit (Thermo Fisher Scientific). Real-time PCR was performed on a Bio-Rad CFX96 machine using SYBR Green mix (Bio-Rad). The relative levels of different transcripts were calculated using the ΔΔCt method, and results were normalized based on the expression of β-actin.

### *In Vitro* Culture of Murine Monocytes

Crude BM cells isolated from C57 BL/6 mice were cultured in RPMI 1640 medium (Sigma-Aldrich) supplemented with 10% FBS (HyClone), 2 mM l-glutamine, 1% penicillin/streptomycin (Thermo Fisher Scientific), and with M-CSF (10 ng ml^−1^) in the presence of different doses of LPS (from 100 to 1 μg ml^−1^). Fresh LPS and M-CSF was added to the cell cultures every 2 days. After 5 days, cells were harvested.

### Flow Cytometry Analyses

Murine BM cells were cultured in the presence of M-CSF and different doses of LPS as described above. In some experiments, control or IRF5 siRNA (30 pmol, Life Technologies) was also added to cell cultures. After 5 days, cells were harvested and stained with anti-CD11b, anti-Ly6C, anti-Ly6G, anti-CCR5, anti-TLR4, and anti-CD14 antibodies (BioLegend). Propidium iodide (PI) was also added to determine the cell viability. To detect the production of IL-12, BM cells cultured for 5 days were treated with PMA (20 ng/ml), ionomycin (1 μg/ml), and GolgiStopTM protein transport inhibitor (BD Biosciences) for 4 h, and then stained with anti-Ly6C, anti-Ly6G, and anti-CD11b antibodies. After fixation and permeabilization using Cytofix/CytopermTM kit (BD Biosciences), cells were stained with anti-IL-12 antibody (BD Biosciences). The cell phenotype was then analyzed by flow cytometer. The data were processed by FACSDiva or Flow Jo.

### Statistical Analysis

Statistical analyses were performed using Prism Version 5 software (GraphPad). Significance of difference was analyzed with a Student’s *t*-test. When more than two groups were compared, one-way ANOVA was performed. Data were presented as means ± SEM. *p* values less than 0.05 were considered significant.

## Author Contributions

RY and SG conducted the research, analyzed the data, and wrote the manuscript. LL designed the research, analyzed the data, and wrote the manuscript.

## Conflict of Interest Statement

The authors declare that the research was conducted in the absence of any commercial or financial relationships that could be construed as a potential conflict of interest.

## References

[1] Yuan R Li L. Dynamic modulation of innate immunity programming and memory. Sci China Life Sci (2016) 59(1):38–43. 10.1007/s11427-015-4998-x 26740103

[2] Morris MC Gilliam EA Li L Innate immune programing by endotoxin and its pathological consequences. Front Immunol (2014) 5:680 10.3389/fimmu.2014.00680 25610440 PMC4285116

[3] Stearns-Kurosawa DJ Osuchowski MF Valentine C Kurosawa S Remick DG The pathogenesis of sepsis. Annu Rev Pathol (2011) 6:19–48. 10.1146/annurev-pathol-011110-130327 20887193 PMC3684427

[4] Lassenius MI Pietilainen KH Kaartinen K Pussinen PJ Syrjanen J Forsblom C Bacterial endotoxin activity in human serum is associated with dyslipidemia, insulin resistance, obesity, and chronic inflammation. Diabetes Care (2011) 34(8):1809–15. 10.2337/dc10-2197 21636801 PMC3142060

[5] Szeto CC Kwan BC Chow KM Lai KB Chung KY Leung CB Endotoxemia is related to systemic inflammation and atherosclerosis in peritoneal dialysis patients. Clin J Am Soc Nephrol (2008) 3(2):431–6. 10.2215/CJN.03600807 18256376 PMC2390956

[6] Yuan R Geng S Chen K Diao N Chu HW Li L. Low-grade inflammatory polarization of monocytes impairs wound healing. J Pathol (2016) 238(4):571–83. 10.1002/path.4680 26690561 PMC4760849

[7] Weiss M Blazek K Byrne AJ Perocheau DP Udalova IA. IRF5 is a specific marker of inflammatory macrophages in vivo. Mediators Inflamm (2013) 2013:245804. 10.1155/2013/245804 24453413 PMC3885211

[8] Chiang MF Yang SY Lin IY Hong JB Lin SJ Ying HY Inducible deletion of the Blimp-1 gene in adult epidermis causes granulocyte-dominated chronic skin inflammation in mice. Proc Natl Acad Sci U S A (2013) 110(16):6476–81. 10.1073/pnas.1219462110 23576729 PMC3631680

[9] Kallies A Carotta S Huntington ND Bernard NJ Tarlinton DM Smyth MJ A role for Blimp1 in the transcriptional network controlling natural killer cell maturation. Blood (2011) 117(6):1869–79. 10.1182/blood-2010-08-303123 21131593

[10] Martins GA Cimmino L Shapiro-Shelef M Szabolcs M Herron A Magnusdottir E Transcriptional repressor Blimp-1 regulates T cell homeostasis and function. Nat Immunol (2006) 7(5):457–65. 10.1038/ni1320 16565721

[11] Chan YH Chiang MF Tsai YC Su ST Chen MH Hou MS Absence of the transcriptional repressor Blimp-1 in hematopoietic lineages reveals its role in dendritic cell homeostatic development and function. J Immunol (2009) 183(11):7039–46. 10.4049/jimmunol.0901543 19915049

[12] Miyauchi Y Ninomiya K Miyamoto H Sakamoto A Iwasaki R Hoshi H The Blimp1-Bcl6 axis is critical to regulate osteoclast differentiation and bone homeostasis. J Exp Med (2010) 207(4):751–62. 10.1084/jem.20091957 20368579 PMC2856022

[13] Salehi S Bankoti R Benevides L Willen J Couse M Silva JS B lymphocyte-induced maturation protein-1 contributes to intestinal mucosa homeostasis by limiting the number of IL-17-producing CD4+ T cells. J Immunol (2012) 189(12):5682–93. 10.4049/jimmunol.1201966 23162130 PMC3529138

[14] Fu Y Glaros T Zhu M Wang P Wu Z Tyson JJ Network topologies and dynamics leading to endotoxin tolerance and priming in innate immune cells. PLoS Comput Biol (2012) 8(5):e1002526. 10.1371/journal.pcbi.1002526 22615556 PMC3355072

[15] Kawai T Akira S. TLR signaling. Semin Immunol (2007) 19(1):24–32. 10.1016/j.smim.2006.12.004 17275323

[16] Takeda K Akira S TLR signaling pathways. Semin Immunol (2004) 16(1):3–9. 10.1016/j.smim.2003.10.003 14751757

[17] Piao W Song C Chen H Diaz MA Wahl LM Fitzgerald KA Endotoxin tolerance dysregulates MyD88- and Toll/IL-1R domain-containing adapter inducing IFN-beta-dependent pathways and increases expression of negative regulators of TLR signaling. J Leukoc Biol (2009) 86(4):863–75. 10.1189/jlb.0309189 19656901 PMC2796624

[18] Lundberg AM Ketelhuth DF Johansson ME Gerdes N Liu S Yamamoto M Toll-like receptor 3 and 4 signalling through the TRIF and TRAM adaptors in haematopoietic cells promotes atherosclerosis. Cardiovasc Res (2013) 99(2):364–73. 10.1093/cvr/cvt033 23417039

[19] Motegi A Kinoshita M Sato K Shinomiya N Ono S Nonoyama S An in vitro Shwartzman reaction-like response is augmented age-dependently in human peripheral blood mononuclear cells. J Leukoc Biol (2006) 79(3):463–72. 10.1189/jlb.0705396 16387840

[20] Slofstra SH ten Cate H Spek CA Low dose endotoxin priming is accountable for coagulation abnormalities and organ damage observed in the Shwartzman reaction. A comparison between a single-dose endotoxemia model and a double-hit endotoxin-induced Shwartzman reaction. Thromb J (2006) 4:13 10.1186/1477-9560-4-13 16930474 PMC1563996

[21] Morris MC Gilliam EA Button J Li L. Dynamic modulation of innate immune response by varying dosages of lipopolysaccharide (LPS) in human monocytic cells. J Biol Chem (2014) 289(31):21584–90. 10.1074/jbc.M114.583518 24970893 PMC4118118

[22] Krausgruber T Blazek K Smallie T Alzabin S Lockstone H Sahgal N IRF5 promotes inflammatory macrophage polarization and TH1-TH17 responses. Nat Immunol (2011) 12(3):231–8. 10.1038/ni.1990 21240265

[23] Sciammas R Davis MM. Modular nature of Blimp-1 in the regulation of gene expression during B cell maturation. J Immunol (2004) 172(9):5427–40. 10.4049/jimmunol.172.9.5427 15100284

[24] Kim SJ Goldstein J Dorso K Merad M Mayer L Crawford JM Expression of Blimp-1 in dendritic cells modulates the innate inflammatory response in dextran sodium sulfate-induced colitis. Mol Med (2014) 20:707–19. 10.2119/molmed.2014.00231 PMC4398669 25826676

[25] Liu Y Wang R Cheng X. [Down-regulated expression of Blimp-1 mRNA in monocytes from patients with active tuberculosis]. Xi Bao Yu Fen Zi Mian Yi Xue Za Zhi (2015) 31(7):949–52. 26146066

[26] Doody GM Care MA Burgoyne NJ Bradford JR Bota M Bonifer C An extended set of PRDM1/BLIMP1 target genes links binding motif type to dynamic repression. Nucleic Acids Res (2010) 38(16):5336–50. 10.1093/nar/gkq268 20421211 PMC2938208

[27] Satpathy S Shenoy GN Kaw S Vaidya T Bal V Rath S Inhibition of terminal differentiation of B cells mediated by CD27 and CD40 involves signaling through JNK. J Immunol (2010) 185(11):6499–507. 10.4049/jimmunol.0903229 20974987

[28] Takeuchi M Rothe M Goeddel DV Anatomy of TRAF2. Distinct domains for nuclear factor-kappaB activation and association with tumor necrosis factor signaling proteins. J Biol Chem (1996) 271(33):19935–42. 10.1074/jbc.271.33.19935 8702708

[29] Chuang HC Sheu WH Lin YT Tsai CY Yang CY Cheng YJ HGK/MAP4K4 deficiency induces TRAF2 stabilization and Th17 differentiation leading to insulin resistance. Nat Commun (2014) 5:4602. 10.1038/ncomms5602 25098764 PMC4143962

[30] Mauro C Crescenzi E De Mattia R Pacifico F Mellone S Salzano S Central role of the scaffold protein tumor necrosis factor receptor-associated factor 2 in regulating endoplasmic reticulum stress-induced apoptosis. J Biol Chem (2006) 281(5):2631–8. 10.1074/jbc.M502181200 16299380

[31] Verfaillie T Salazar M Velasco G Agostinis P. Linking ER stress to autophagy: potential implications for cancer therapy. Int J Cell Biol (2010) 2010:930509. 10.1155/2010/930509 20145727 PMC2817393

[32] Shevlin E Miggin SM. The TIR-domain containing adaptor TRAM is required for TLR7 mediated RANTES production. PLoS One (2014) 9(9):e107141. 10.1371/journal.pone.0107141 25211222 PMC4161432

[33] Netea MG Quintin J van der Meer JW. Trained immunity: a memory for innate host defense. Cell Host Microbe (2011) 9(5):355–61. 10.1016/j.chom.2011.04.006 21575907

[34] Gordon S The macrophage: past, present and future. Eur J Immunol (2007) 37(Suppl 1):S9–17. 10.1002/eji.200737638 17972350

[35] Deng H Maitra U Morris M Li L. Molecular mechanism responsible for the priming of macrophage activation. J Biol Chem (2013) 288(6):3897–906. 10.1074/jbc.M112.424390 23264622 PMC3567643

[36] Cohen M Matcovitch O David E Barnett-Itzhaki Z Keren-Shaul H Blecher-Gonen R Chronic exposure to TGFbeta1 regulates myeloid cell inflammatory response in an IRF7-dependent manner. EMBO J (2014) 33(24):2906–21. 10.15252/embj.201489293 25385836 PMC4282639

[37] Wesche H Henzel WJ Shillinglaw W Li S Cao Z. MyD88: an adapter that recruits IRAK to the IL-1 receptor complex. Immunity (1997) 7(6):837–47. 10.1016/S1074-7613(00)80402-1 9430229

[38] Subramanian M Thorp E Hansson GK Tabas I. Treg-mediated suppression of atherosclerosis requires MYD88 signaling in DCs. J Clin Invest (2013) 123(1):179–88. 10.1172/JCI64617 23257360 PMC3533292

[39] Xi CX Xiong F Zhou Z Mei L Xiong WC. PYK2 interacts with MyD88 and regulates MyD88-mediated NF-kappaB activation in macrophages. J Leukoc Biol (2010) 87(3):415–23. 10.1189/jlb.0309125 19955209 PMC2830122

[40] Broad A Jones DE Kirby JA Toll-like receptor (TLR) response tolerance: a key physiological “damage limitation” effect and an important potential opportunity for therapy. Curr Med Chem (2006) 13(21):2487–502. 10.2174/092986706778201675 17017906

[41] Morris M Li L. Molecular mechanisms and pathological consequences of endotoxin tolerance and priming. Arch Immunol Ther Exp (Warsz) (2012) 60(1):13–8. 10.1007/s00005-011-0155-9 22143158

[42] Salagianni M Galani IE Lundberg AM Davos CH Varela A Gavriil A Toll-like receptor 7 protects from atherosclerosis by constraining “inflammatory” macrophage activation. Circulation (2012) 126(8):952–62. 10.1161/CIRCULATIONAHA.111.067678 22787112

[43] Broad A Kirby JA Jones DE Applied Immunology and Transplantation Research Group. Toll-like receptor interactions: tolerance of MyD88-dependent cytokines but enhancement of MyD88-independent interferon-beta production. Immunology (2007) 120(1):103–11. 10.1111/j.1365-2567.2006.02485.x 17034424 PMC2265871

[44] Liu B Liu Q Yang L Palaniappan SK Bahar I Thiagarajan PS Innate immune memory and homeostasis may be conferred through crosstalk between the TLR3 and TLR7 pathways. Sci Signal (2016) 9(436):ra70. 10.1126/scisignal.aac9340 27405980 PMC5087126

