# Supplementary material for: Molecular Mechanisms That Underlie the Dynamic Adaptation of Innate Monocyte Memory to Varying Stimulant Strength of TLR Ligands
Source: Front Immunol. 2016 Nov 10;7:497. doi: 10.3389/fimmu.2016.00497 (PMC5103159; doi:10.3389/fimmu.2016.00497)
Supplement: Supplementary file 1 [file presentation_1.pdf]

Supplementary Figures

Molecular mechanisms that underlie the dynamic adaptation of innate monocyte memory to varying stimulant strength of TLR ligands

Ruoxi Yuan, [1, 2] , Shuo Geng[1], Liwu Li, [1, 2, *]

[1] Department of Biological Sciences, Virginia Polytechnic Institute and State University, Blacksburg, VA 24061, USA

[2] Department of Biomedical Sciences & Pathobiology, Virginia-Maryland College of Veterinary Medicine, Virginia Polytechnic Institute and State University, Blacksburg, VA 24061, USA

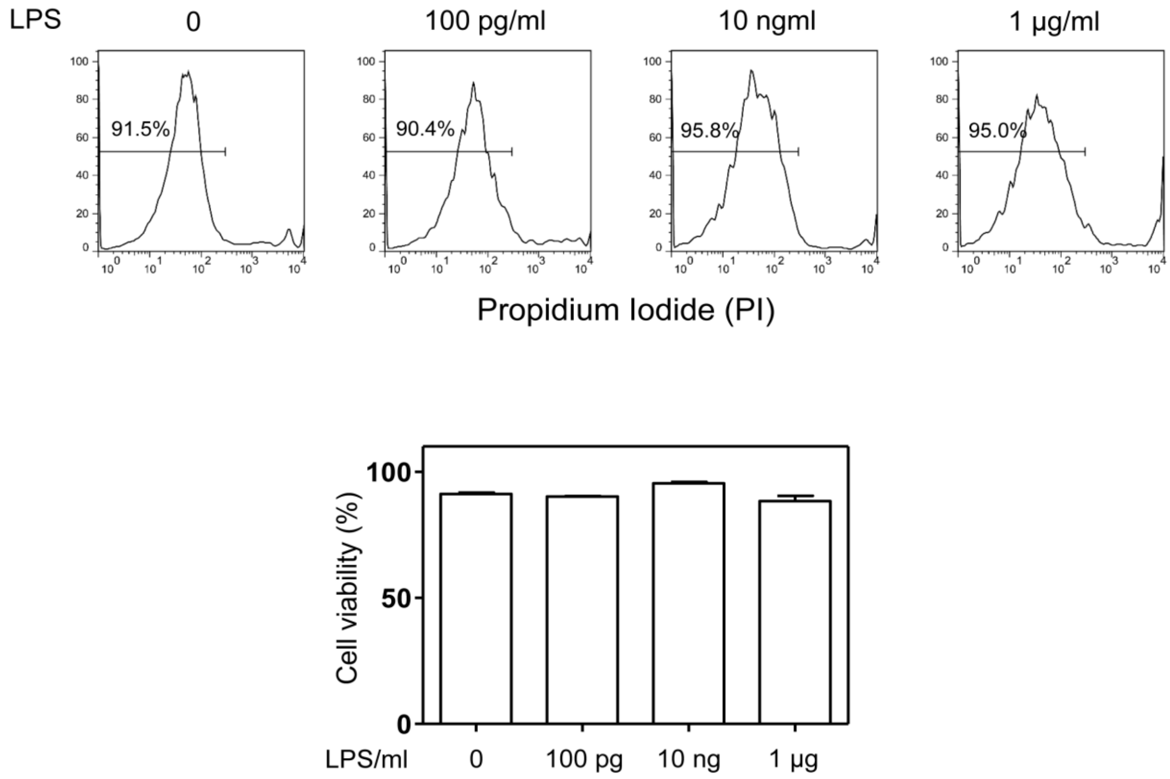

Supplementary Figure S1. Cell viability measurement of co-cultured monocytes with varying dosages of LPS and M-CSF. BM cells were cultured with M-CSF (10 ng/ml) and different doses of LPS for 5 days, and then stained with PI. Cell viability was determined by flow cytometry. Error bar represents s.e.m. of three experiments.

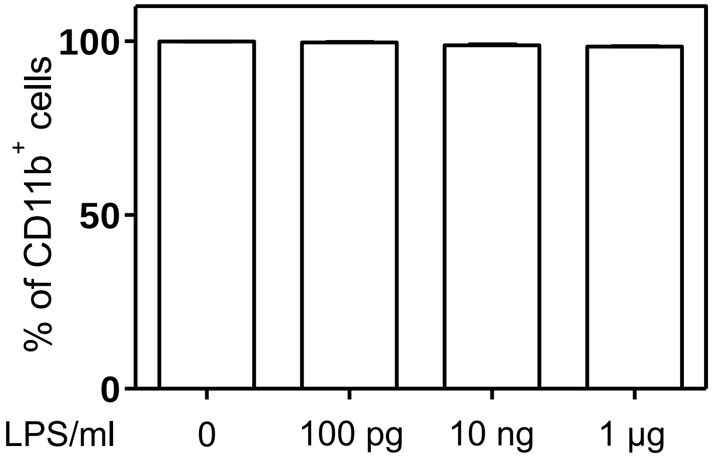

Supplementary Figure S2. Cell surface levels of CD11b measurement of cultured monocytes with M-CSF. BM cells were cultured with M-CSF (10 ng/ml) and different doses of LPS for 5 days, and then stained with anti-CD11b antibody and PI. Percentages of CD11b$^+$ cells within viable cells was analyzed by flow cytometry. Data represents three experiments.

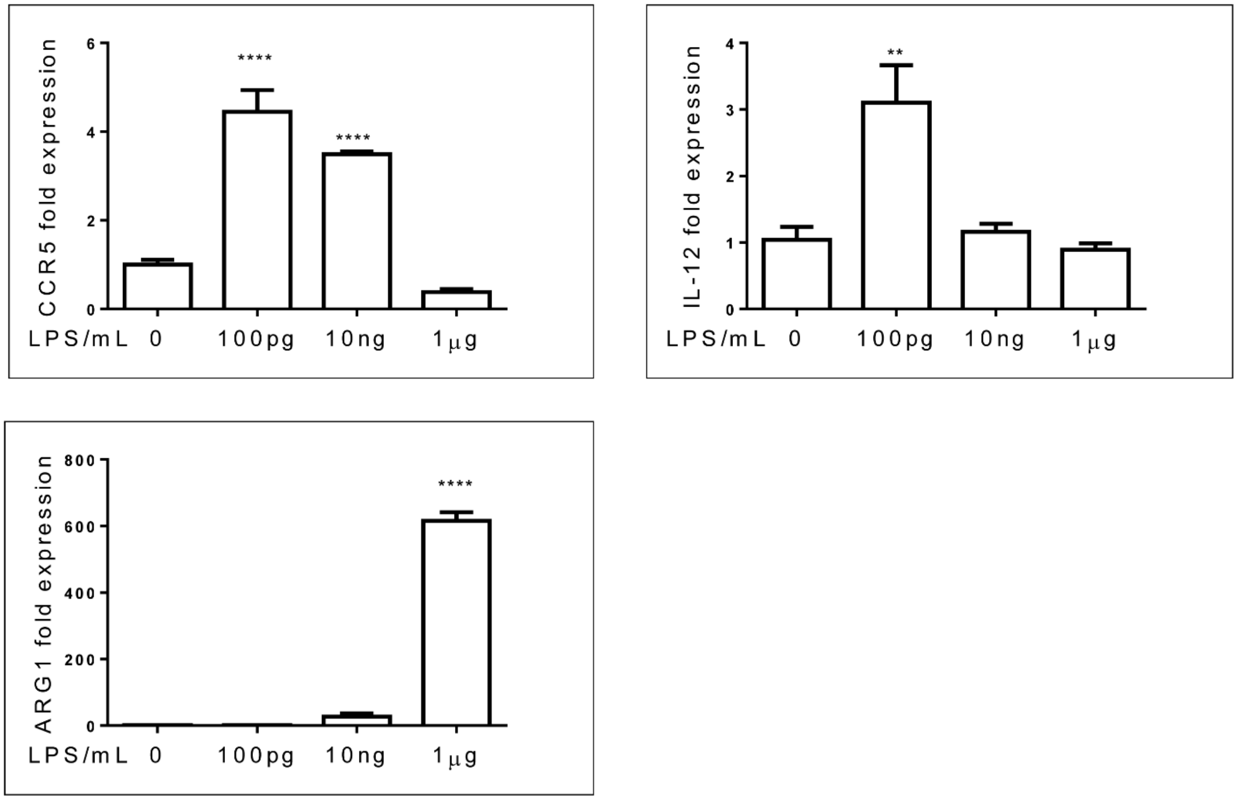

Supplementary Figure S3. Additional experiments demonstrating the differential modulation of selected genes by varying dosages of LPS. Total RNA was isolated from monocytes treated with different dosages of LPS for 5 days. Real-time PCR was performed to determine the expression levels of CCR5 (A), IL-12 (B), ARG1 (C) and iNOS (D). Data are representative of three separate experiments (error bar represent s.e.m., **, p<0.05, ****, p<0.0005; as compared to non-treated control group, student t test).

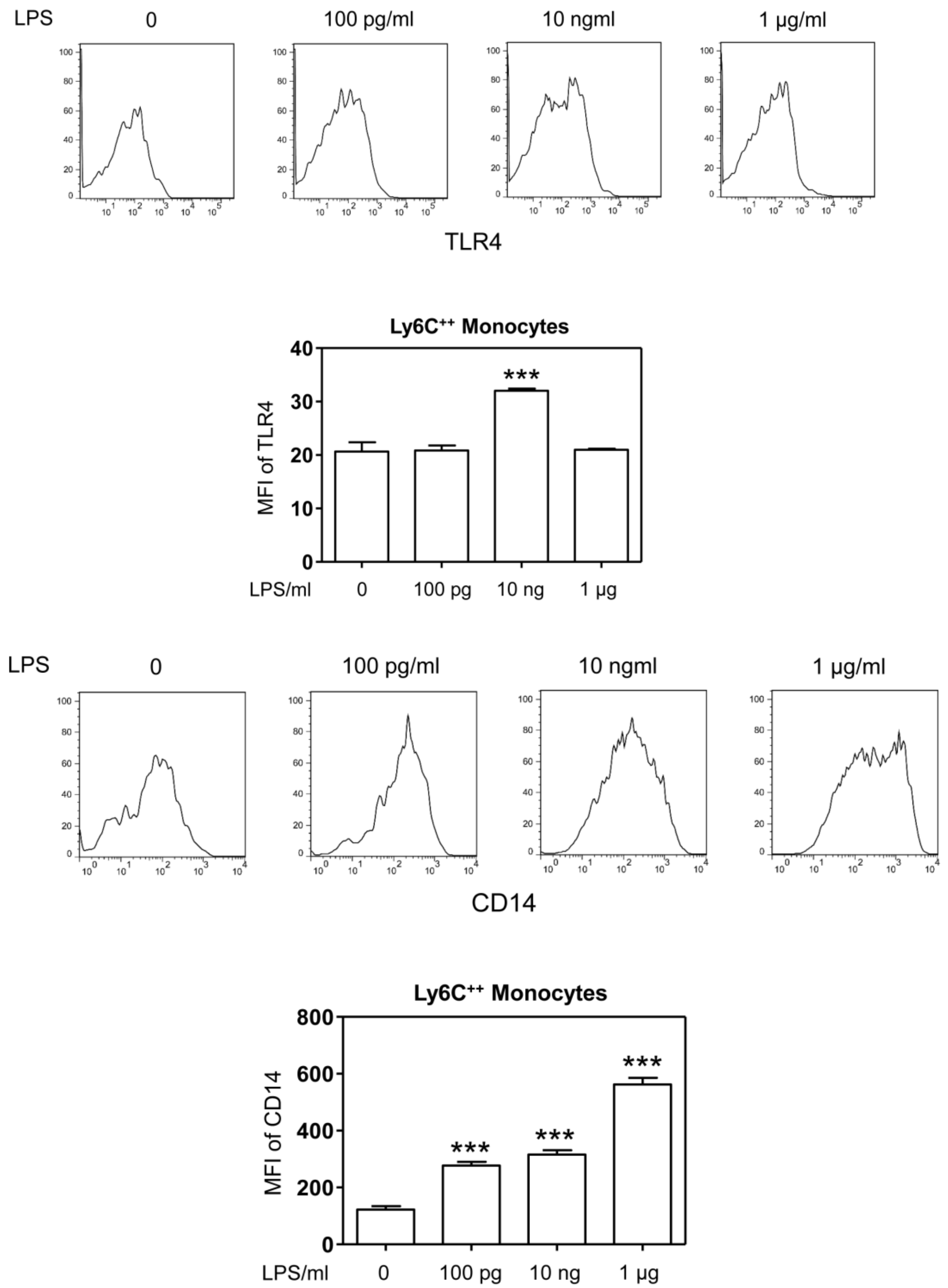

Supplementary Figure S4. Cell surface levels of TLR4 and CD14 measurement of co-cultured monocytes with varying dosages of LPS and M-CSF. BM cells were cultured with M-CSF (10 ng/ml) and different doses of LPS for 5 days. Cell surface expression of TLR4 and CD14 within monocytes was detected by flow cytometry. Error bar represents s.e.m. of three experiments. (***, p<0.001; as compared to non-treated control group, student t test).
